# Supplementary material for: tbiExtractor: A framework for extracting traumatic brain injury common data elements from radiology reports
Source: PLoS One. 2020 Jul 1;15(7):e0214775. doi: 10.1371/journal.pone.0214775 (PMC7329124; doi:10.1371/journal.pone.0214775)
Supplement: S1 Appendix — (DOCX) [file pone.0214775.s001.docx]

**Supplementary 1. CLASSIFY-TBI Study Design**

| Enrollment Period | May 2016 – June 2018 |
| --- | --- |
| Institution | Hennepin Healthcare, Level 1 Trauma Center  Minneapolis, MN |
| Patient Groups | Patients presenting with *traumatic brain injuries* (e.g., patient went head first through windshield during a motor vehicle crash) or *non-traumatic brain injuries* (e.g., patient experienced prolonged oxygen deprivation from an allergic reaction resulting in anoxic brain injury). |
| Inclusion Criteria | - Between 4-100 years of age - Clinically ordered non-contrast head CT on admission |
| Exclusion Criteria | - Injury occurred more than 6 hours before emergency department presentation - Time of injury cannot be reliably determined - Part of an interventional trial that could affect the validity of the assessment - Head trauma within the past 6 months to current assessment - Prisoner, person in custody, or ward of the state - Presentation to special care unit of emergency department - Neurodevelopmentally abnormal - Major psychiatric and/or neurological disorders - Unable to speak and understand English |
