# Supplementary material for: tbiExtractor: A framework for extracting traumatic brain injury common data elements from radiology reports
Source: PLoS One. 2020 Jul 1;15(7):e0214775. doi: 10.1371/journal.pone.0214775 (PMC7329124; doi:10.1371/journal.pone.0214775)
Supplement: S2 Appendix — (DOCX) [file pone.0214775.s002.docx]

**Supplementary 2. Calculate TF-IDF and Cosine Similarities**

Using scikit-learn[27] TfidfVectorizer, the corpus was converted into a matrix of TF-IDF (term-frequency times inverse document-frequency) features using *n*-grams with *n*-range from one to ten. Each radiology report is considered one document in the corpus. To calculate the TF-IDF matrix, a list of unique terms in the corpus is made. Then each document is quantified by the frequency of unique terms, meaning if there are *k* unique terms in the corpus, each document will be a vector of length *k* with numerical counts for each vector component. Next, to mitigate the effects of verbose documents and irrelevant but frequently occurring terms, the term-frequency is adjusted by the inverse document-frequency to enhance rare terms and discount terms frequently represented in the corpus. With *d* radiology report documents, the result is a *d x k* TF-IDF matrix, which is normalized by the Euclidean norm to ensure each vector is of unit length.

Cosine similarities are then calculated between each pair of radiology reports by multiplying the TF-IDF matrix by its transpose, resulting in a *d x d* symmetric matrix. Note, cosine similarity quantifies the closeness of two documents by taking the cosine of the angle between two vectors. If the angle between the vectors is small, the cosine similarity will tend towards one, indicating a high similarity between the two documents. Conversely, if the angle between the vectors is large, the cosine similarity will tend towards zero, indicating low similarity between the two documents.

Using the cosine similarity for each pair of radiology reports, one radiology report was randomly selected and all radiology reports with at least 0.70 cosine similarity to that radiology report were collected in a set. From this set, one radiology report was randomly selected to keep for further analysis and the remainder were removed. This was applied recursively for each set until each radiology report was retained for further analysis or marked for removal. If a radiology report did not have another radiology report with at least 0.70 cosine similarity, it was automatically retained for further analysis. The purpose of this removal of radiology reports from the dataset was to reduce the data requiring human annotation. By using cosine similarities between two radiology reports, if two reports are nearly identical, annotating one of them is sufficient.
