# Supplementary material for: tbiExtractor: A framework for extracting traumatic brain injury common data elements from radiology reports
Source: PLoS One. 2020 Jul 1;15(7):e0214775. doi: 10.1371/journal.pone.0214775 (PMC7329124; doi:10.1371/journal.pone.0214775)

**Supplementary 3. Radiology Report Annotation GUI Screenshot.** This screenshot represents the screen an annotator would see when beginning a new annotation. The plain text of the neuroradiologist’s report findings was presented in the top section of the GUI without access to the CT images. This was done to ensure only the documented findings from the board-certified neuroradiologist were interpreted. In the bottom section of the GUI, all 27 lexical targets and their baseline modifier options (i.e., ABSENT or NORMAL), chosen to represent healthy anatomy, are shown. As the annotator evaluated the radiology report text, any findings which deviated from normal anatomy had their modifier updated from the drop-down menu to accurately reflect the pathological status (i.e., PRESENT, SUSPECTED, INDETERMINATE, NOT SPECIFIED, or ABNORMAL).


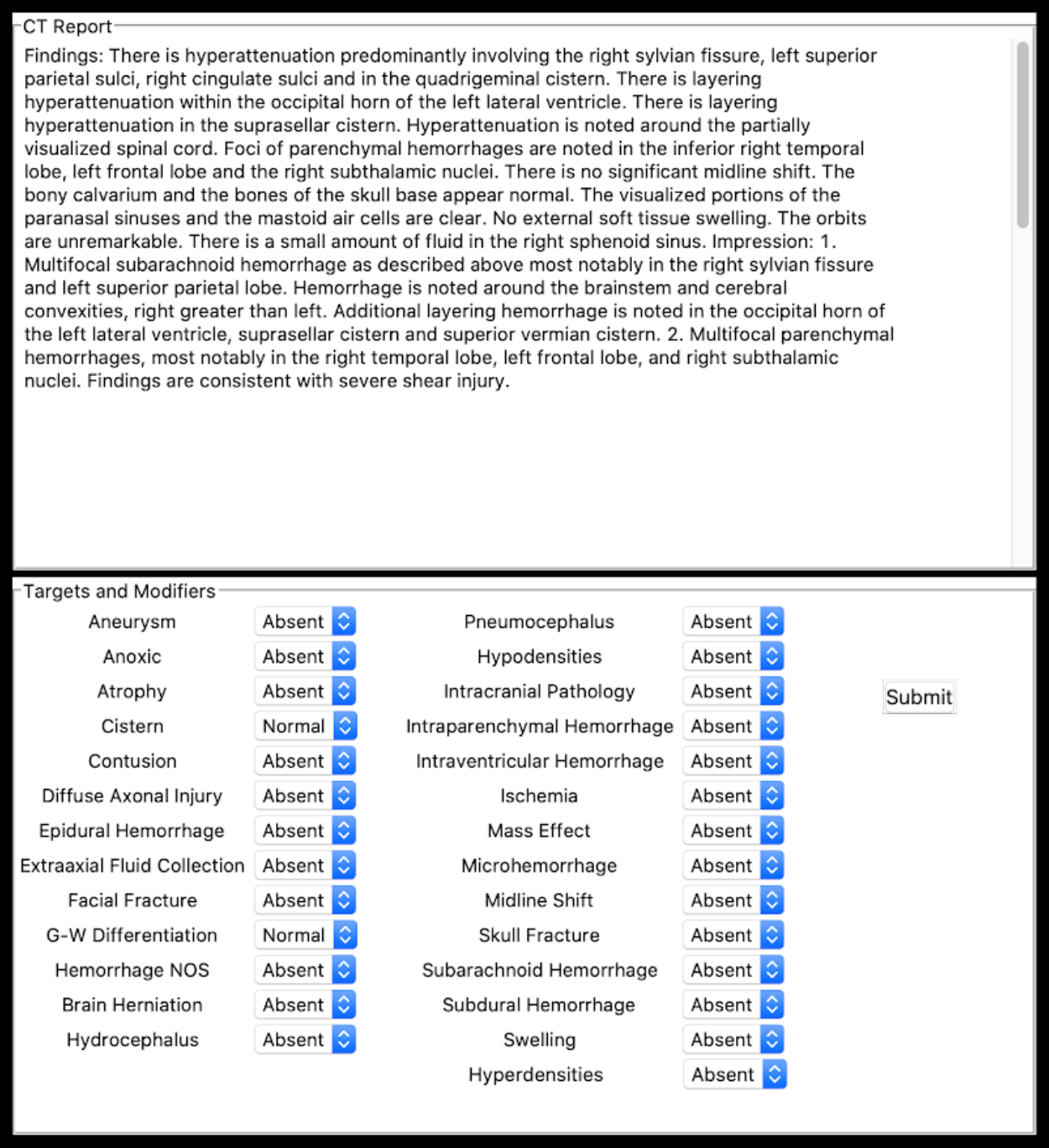

Supplement: S3 Appendix — (DOCX) [file pone.0214775.s003.docx]
