# Supplementary material for: tbiExtractor: A framework for extracting traumatic brain injury common data elements from radiology reports
Source: PLoS One. 2020 Jul 1;15(7):e0214775. doi: 10.1371/journal.pone.0214775 (PMC7329124; doi:10.1371/journal.pone.0214775)
Supplement: S4 Appendix — (PDF) [file pone.0214775.s004.pdf]

S4. Classification Performance Metrics Equations

Sensitivity = $\frac{TP}{TP+FN}$

Specificity = $\frac{TN}{TN+FP}$

Positive Predictive Value = $\frac{TP}{TP+FP}$

Negative Predictive Value = $\frac{TN}{TN+FN}$

Accuracy = $\frac{TP+TN}{TP+FP+TN+FN}$

F1 Score = $\frac{2*PPV*Sensitivity}{PPV+Sensitivity}$
